# Infantile GM1 Gangliosidosis with Epilepsy Associated with a Same-Codon GLB1 Variant (c.808T>G/c.808T>C)

**DOI:** 10.3390/genes17060691

**Published:** 2026-06-12

**Authors:** Rimma Gamirova, Arina Grishagina, Elena Gorobets, Giuditta Bargiacchi, Marco Carotenuto

**Affiliations:** 1 Neurocognitive Research Laboratory, Department of Neurology with Courses in Psychiatry, Clinical Psychology, and Medical Genetics, Institute of Fundamental Medicine and Biology, Kazan (Volga Region) Federal University, 18 Kremlevskaya St., Kazan 420008, Russia; arinaleznina@gmail.com; 2 Neurocognitive Research Laboratory, Department of Applied and Experimental Linguistics, Center for Speech Pathology, Kazan (Volga Region) Federal University, 18 Kremlevskaya St., Kazan 420008, Russia; elena.gorobets@kpfu.ru; 3 Clinic of Child and Adolescent Neuropsychiatry, Department of Mental Health, Physical and Preventive Medicine, University of Campania “Luigi Vanvitelli”, 80131 Naples, Italy; giuditta.bargiacchi@studenti.unicampania.it (G.B.); marco.carotenuto@unicampania.it (M.C.)

**Keywords:** GM1 gangliosidosis, GLB1, β-galactosidase, codon 270, epilepsy, genotype–phenotype correlation

## Abstract

GM1 gangliosidosis is an autosomal recessive lysosomal storage disorder caused by a deficiency of β-galactosidase due to pathogenic variants in the *GLB1* gene. Almost 300 pathogenic or likely pathogenic variants have been identified, associated with a phenotypic spectrum ranging from GM1 gangliosidosis to mucopolysaccharidosis type IVB. Disease severity is largely determined by the extent to which specific variants impair enzymatic catalytic activity, particularly through disruption of substrate recognition and binding within the active site. We report a patient with GM1 gangliosidosis type I harboring two pathogenic missense variants, c.808T>G (p.Tyr270Asp) and c.808T>C (p.Tyr270His), in a compound heterozygous state. To the best of our knowledge, this specific allelic combination has not been previously described. Both variants affect the same codon, resulting in distinct amino acid substitutions at position 270, a residue critically involved in maintaining the structural and functional integrity of the catalytic domain of β-galactosidase. Disruption at this site is expected to severely compromise enzymatic activity. Comparative analysis with previously reported cases carrying variants at the same residue, in either homozygous or compound heterozygous states, demonstrates a possible consistent association with the infantile form of GM1 gangliosidosis, characterized by a rapidly progressive neurodegenerative course and multisystem involvement. Collectively, these findings provide additional support for the hypothesis that codon 270 can be regarded as a critical functional hotspot within *GLB1*, where even distinct amino acid substitutions can result in profound enzymatic dysfunction and a severe early-onset phenotype.

## 1. Introduction

GM1 gangliosidosis is a rare hereditary lysosomal storage disorder with autosomal recessive inheritance, caused by deficiency of β-galactosidase, an enzyme required for the degradation of complex carbohydrates associated with structurally diverse molecules, including GM1 ganglioside and keratan sulfate [1,2,3,4]. The estimated incidence of GM1 gangliosidosis ranges from 1:100,000 to 1:200,000 live births [1,3,4], although specific phenotypic variants appear to cluster in defined geographic regions, with increased prevalence reported in Southern Brazil, Japan, and within the Roma population [5].

Pathogenic variants in the *GLB1* gene (3p22), encoding β-galactosidase, are responsible for both GM1 gangliosidosis and mucopolysaccharidosis type IVB [2]. β-galactosidase is a lysosomal hydrolase involved in the cleavage of terminal β-galactose residues from GM1 ganglioside. Reduced or absent enzymatic activity leads to progressive accumulation of GM1 ganglioside and related glycoconjugates within lysosomes across multiple tissues, with predominant involvement of the central nervous system [2]. The phenotypic expression of *GLB1* variants depends on their structural and functional impact on the enzyme. Variants affecting regions closer to the ligand-binding site and β-domain 2 are more commonly associated with mucopolysaccharidosis type IVB, resulting in partial enzymatic dysfunction and preferential impairment of keratan sulfate degradation. In contrast, mutations involving the core protein and the TIM-barrel domain—critical for catalytic activity—are typically associated with GM1 gangliosidosis and more severe enzymatic impairment [6,7].

Accumulation of GM1 ganglioside in the central nervous system (CNS) triggers a cascade of pathogenic mechanisms, including neuronal apoptosis [8,9], progressive demyelination [10], disruption of synaptic transmission [11,12], and chronic neuroinflammation, ultimately driving disease progression [13].

GM1 gangliosidosis is characterized by marked clinical heterogeneity and variable age of onset, largely reflecting the degree of residual β-galactosidase activity determined by the underlying *GLB1* variants in homozygous or compound heterozygous states [2,14,15]. Based on age at onset and clinical severity, three main forms are recognized.

Type I (infantile form) typically presents within the first 6 months of life and is characterized by a severe and rapidly progressive neurodegenerative course. Early manifestations include diffuse hypotonia and delayed psychomotor development, followed by spasticity, rigidity, dysphagia, dystonia, ataxia, and progressive cognitive decline, often accompanied by hearing and visual impairment [16,17,18,19]. Epilepsy represents a frequent and prominent feature [19,20,21], with electroencephalographic findings including diffuse slowing of background activity and/or multifocal epileptiform discharges [21,22]. Systemic involvement is common and includes hepatosplenomegaly, cardiomyopathy, coarse facial features, and retinal “cherry-red spot” [1,21]. The prognosis is poor, with death typically occurring within the first 1–2 years of life [21,23,24].

Type II (late infantile/juvenile form) usually manifests between 7 months and 3 years of age and is characterized by progressive hypotonia, dystonia, ataxia, dysphagia, epilepsy, motor regression, and cognitive decline [21,23,24,25,26]. Visceral involvement is less prominent, and hepatomegaly and cardiomyopathy are less frequently observed [2]. Disease progression is slower compared to the infantile form, with survival extending into adolescence or early adulthood [21].

Type III (adult form) presents later in life and is characterized by a milder but progressive course, including extrapyramidal symptoms, corneal opacities, muscle hypotonia, and cognitive impairment [24,27,28].

Diagnosis is based on the demonstration of reduced β-galactosidase activity and molecular genetic analysis to identify pathogenic variants in the *GLB1* gene [27]. Currently, management remains largely supportive; however, several therapeutic strategies are under investigation, including enzyme replacement therapy, substrate reduction therapy, hematopoietic stem cell transplantation, pharmacological chaperones, and gene therapy approaches based on adeno-associated viral vectors [29,30,31,32,33].

## 2. Case Report

We report a 3-year-old girl with early infantile GM1 gangliosidosis (type I) presenting with severe developmental and drug-resistant epilepsy characterized by frequent, polymorphic seizures and rapid neurodevelopmental regression. Written informed consent has been obtained from the patient’s parents to publish this paper.

At the age of 3 years old, for the first time the girl was examined by an epileptologist due to bilateral tonic–clonic seizures with perioral cyanosis and stertorous breathing lasting up to 1–2 min several times a day (some of them with the development of status epilepticus), frequent myoclonic seizures during the day and focal alternating motor clonic seizures lasting up to 1–2 min with a frequency of up to 1–2 times a day.

The girl had a 2.5-year history of progressive neurodevelopmental regression, involving motor, language, and social domains.

*Ante/perinatal history*. The pregnancy was achieved via in vitro fertilization and was complicated during the second trimester by nausea and vomiting, rhinovirus infection, and abruptio placentae. Prenatal ultrasound at 24 weeks revealed ventriculomegaly. Delivery occurred spontaneously at term (40 weeks), with a birth weight of 4450 g (macrosomia) and Apgar scores of 7 and 8 at 1 and 5 min, respectively.

*Early development* was reportedly normal until approximately 3 months of age, after which progressive stagnation and subsequent regression became evident.

At 3 months of age, neurological assessment revealed significant diffuse muscle hypotonia and hypertensive hydrocephalus, confirmed by neurosonography showing enlargement of subarachnoid cerebrospinal fluid spaces. Initial metabolic screening, including aminoacidopathies, organic acidurias, and mitochondrial beta-oxidation defects, was unremarkable.

The first myoclonic jerks while awake appeared at 6–7 months. Electroencephalography (EEG) *at 11 months* revealed slowing of the cortical activity and regional bioccipital slowing, but epileptiform discharges were not registered. Brain magnetic resonance imaging (MRI) performed at 11 months revealed delayed myelination, moderate ventriculomegaly, expansion of frontal subarachnoid spaces, and the presence of cavum veli interpositi. Additional findings included mild periventricular leukomalacia, likely secondary to hypoxic–ischemic injury, and mild hypoplasia of the cerebellum and corpus callosum (Figure 1 and Figure 2). Subsequent clinical evaluation at 7 months documented global developmental delay, generalized hypotonia, and alternating exotropia. Laboratory investigations showed elevated systemic enzymes (AST 91 U/L, LDH 750 U/L, ALP 813 U/L).

*At 11 months*, the initial lysosomal screening was within normal limits, except for a non-specific elevation of hexosylsphingosine (171.93 ng/mL; reference range 0.5–10 ng/mL). Further metabolic investigations at 12 months, including very long-chain fatty acids, mitochondrial DNA analysis, urinary organic acids, and testing for Prader–Willi and Angelman syndromes, were unremarkable.

*At 17 months*, repeat lysosomal enzyme analysis revealed a moderate to severe deficiency in beta-galactosidase activity (27.73 μM/L/h; reference range 50–200 μM/L/h), raising suspicion for GM1 gangliosidosis.

Whole-exome sequencing identified two pathogenic variants in the *GLB1* gene in compound heterozygosity: c.808T>G (p.Tyr270Asp) and c.808T>C (p.Tyr270His). Sanger sequencing confirmed parental segregation, with the father carrying the p.Tyr270His variant and the mother the p.Tyr270Asp variant, confirming that the two variants are located in trans and consistent with autosomal recessive inheritance.

Notably, both variants affect the same codon (Tyr270), suggesting a potential mutational hotspot with functional relevance for enzyme activity.

*Following genetic diagnosis at 23 months*, clinical re-evaluation revealed specific dysmorphic features, including a prominent convex forehead, hypertelorism, epicanthal folds, a depressed nasal bridge, low-set ears, a short philtrum, a hydrocephalic skull, and dental anomalies.

Video-EEG monitoring at 2 years demonstrated diffuse cortical slowing, diffuse and regional epileptiform discharges (polyspike-slow wave) predominantly in the frontal-central areas, mostly from the right (Figure 3).

Neurological examination showed severe global impairment, including altered level of consciousness, axial hypotonia, distal spasticity, and pyramidal signs. Brain MRI confirmed diffuse cerebral atrophy (Figure 4).

*At 30 months*, there were registered alternating focal clonic motor seizures in the limbs with subsequent transformation into bilateral tonic–clonic seizures and the development of status epilepticus.

Antiseizure treatment with levetiracetam and clobazam (administered at optimized weight-adjusted dosages: 40 mg/kg a day and 0,5 mg/kg a day, respectively) resulted in partial control of both focal and myoclonic seizures, without achieving seizure freedom.

The clinical picture was consistent with a severe developmental phenotype with focal motor, myoclonic and bilateral tonic–clonic epileptic seizures with a tendency to frequent epileptic status (which required the administration of benzodiazepines).

Ophthalmological evaluation revealed a cherry-red spot, further supporting the diagnosis.

The integrated clinical, biochemical, neuroimaging, and genetic findings confirmed the diagnosis of early infantile GM1 gangliosidosis (type I).

The phenotype was characterized by rapid neurodevelopmental regression, severe developmental and epileptic encephalopathy, spastic tetraparesis, drug-resistant epilepsy, and specific ophthalmological findings.

Importantly, the absence of dysostosis multiplex in this patient highlights the marked phenotypic variability of GM1 gangliosidosis and underscores that its absence should not delay or preclude diagnosis in the presence of suggestive clinical and biochemical findings.

## 3. Nucleotide Variants c.808T>G and c.808T>C in the *GLB1* Gene

According to the ClinVar database (April 2026), the nucleotide variant c.808T>G in the *GLB1* gene (NM_000404.4(*GLB1*): c.808T>G (p.Tyr270Asp), Variation ID: 284172) is pathogenic/probably pathogenic and associated with the development of GLB1-associated diseases (GM1-gangliosidosis and mucopolysaccharidosis type IVB). Table 1 summarizes the results of the search for published research results and/or clinical cases associated with the nucleotide variant NM_000404.4(*GLB1*):c.808T>G. The missense mutation c.808T>G (p.Tyr270Asp) was first described in [34] in 2001 as new in one of the studied patients in a compound heterozygous state (genotype: p.Thr82Met/p.Tyr270Asp).

Moreover, Hofer et al. described the nucleotide variant c.808T>G (p.Tyr270Asp) in 14% of patients with alleles examined and noted that, except for one patient, all patients had type 1 GM1-gangliosidosis. Two patients were homozygous for mutation c.808T>G (p.Tyr270Asp), and three were compound heterozygotes with genotypes p.Tyr270Asp/c.1601_1602insGCCA, p.Y270D/p.K346N, p.Y270D/p.T82M, respectively [35]. The result of this mutation is that tyrosine at position 270 is located near the most important catalytic centers of the enzyme beta-galactosidase, so a missense mutation and amino acid substitution of a tyrosine residue lead to a significant impairment of the functional activity of the protein, with the frequent development of GM1-gangliosidosis type 1 [39,40]. Bychkov et al. in 2022 described a clinical case of mucopolysaccharidosis IVB, with a c.808T>G (p.Tyr270Asp) variant in *GLB1* in a heterozygous state, together with the insertion of a processed pseudogene in *GLB1*. This insertion affects the arrangement of components of the protein beta-structure, which contains regions necessary for the enzyme’s catalytic activity, and probably significantly disrupts the activity of β-galactosidase (β-GAL) [38].

The nucleotide variant c.808T>C (NM_000404.4(*GLB1*): c.808T>C (p.Tyr270His) has been described in the ClinVar database (Variation ID: 1330373) (April 2026); however, there is only one record indicating that this nucleotide variant is likely associated with GM1-gangliosidosis and the formal ACMG classification is not provided. Komissarova et al. in 2023 described a clinical case of carrying c.807T>C (p.Tyr270His) in a heterozygous woman, whose daughter is homozygous for c.807T>C (p.Tyr270His) and has a severe neurodegenerative disease. The authors associate the nucleotide variant c.808T>C (p.Tyr270His) of the *GLB1* gene in a heterozygous state with the cause of severe stroke in a patient [41]; however, a causal relationship between heterozygous carrier status and stroke remains suppositive and should be interpreted with caution. The nucleotide variant c.808T>C (p.Tyr270His), as well as c.808T>G (p.Tyr270Asp), refers to a missense mutation. Since the position of the initial amino acid residue tyrosine is extremely important for the normal functioning of the enzyme, it can be assumed that its replacement by histidine c.808T>C (p.Tyr270His) also leads to impaired functional activity of the protein. The availability of only a single previously reported case represents a limitation. However, the information derived from that case remains important and, together with our findings, contributes to the interpretation of c.808T>C in the context of GM1 gangliosidosis.

The comparative characteristics of patients exhibiting the c.808T>G (p.Tyr270Asp) variant in *GLB1* and diagnosed with either GM1-gangliosidosis or mucopolysaccharidosis IVB are presented in Table 2.

## 4. Discussion

The p.Tyr270Asp (c.808T>G) variant in *GLB1* may contribute substantially to disease severity, showing a strong association with the infantile form of GM1 gangliosidosis. Available evidence indicates that both homozygous cases and the majority of compound heterozygous carriers of this variant present with a severe clinical phenotype. This genotype–phenotype relationship is consistent with the marked impairment of β-galactosidase activity caused by the non-conservative substitution of tyrosine with aspartic acid, which alters both charge and structural properties of the residue, leading to severe disruption of enzymatic function [35,39,40,42]. However, the genotype–phenotype correlation observed in these cases should be interpreted with caution. Additional factors, including prenatal or perinatal events and other genetic modifiers, may have contributed to the clinical severity. Further studies with detailed clinical and biochemical phenotyping are required to validate and refine this correlation.

As summarized in Table 2, reported substitution at codon 270 (p.Tyr270Asp) is associated with markedly reduced β-galactosidase activity and a moderate to severe phenotype. This pathogenic variant at a single residue supports the functional importance of codon 270 and is consistent with its designation as a potential functional hotspot. Consistent with this, patients harboring the p.Tyr270Asp appear to exhibit a severe neurodegenerative phenotype. Notably, Paschke et al. (2001) initially described a patient with the genotype p.Thr82Met/p.Tyr270Asp as affected by Morquio type B disease; however, long-term follow-up revealed progressive neurological deterioration, including complete loss of speech and development of spastic tetraparesis, indicating central nervous system involvement not characteristic of mucopolysaccharidosis type IVB [34]. In our patient, delayed motor development became evident at 6 months, followed by progressive regression of motor skills from 14 months of age, a trajectory consistent with the early-onset neurodegenerative phenotype of GM1 gangliosidosis type I. Moreover, analysis of the cases reported in Table 2 indicates that visceral involvement, including hepatosplenomegaly and cardiac manifestations, is present in the majority of patients. The occurrence of epileptic seizures in GM1 gangliosidosis warrants particular attention. Although seizures have not been consistently reported in patients harboring the p.Tyr270Asp variant, they represented a prominent clinical feature in our case, with high daily seizure frequency. Notably, epilepsy is recognized as a common and clinically relevant manifestation of GM1 gangliosidosis.

In 2019, Arash-Kaps et al. reported that epilepsy occurs in up to 59% of patients, particularly in the infantile form [17]. Seizure types most frequently include generalized tonic seizures, although hypomotor and myoclonic seizures have also been described. EEG records irregular slowing of background activity and/or multiregional epileptiform discharges [21]. Electroencephalographic findings typically show diffuse slowing of background activity and/or multifocal epileptiform discharges, with persistent low-frequency irregular activity considered a pathological hallmark in both GM1 and GM2 gangliosidoses [21,34,43]. These observations are consistent with the neurophysiological data obtained in our patient, whose EEG showed marked diffuse slowing of cortical rhythms, regional slowing in the occipital areas, regional and diffuse polyspike-slow-wave epileptiform activity, which is often associated with myoclonic seizures.

With regard to the biochemical enzyme assay, β-galactosidase activity was measured in different specimen types, including fibroblasts, leukocytes, lymphoblasts, and dried blood spots, the standardized fluorimetric method with 4-methylumbelliferyl-β-D-galactopyranoside (4-MU) as a synthetic substrate was used in all reviewed cases [34,35,36,37,38]. Despite non-uniform reference ranges across studies, a consistent pattern of decreased enzyme deficiency was observed in all patients, including our own case. Detailed values are provided in Table 2. In our case, the enzyme residual activity is approximately 55% of the lower reference limit and is higher than the near-zero values often cited for classic infantile GM1 gangliosidosis, but it should be noted that enzyme activity measured in vitro does not always correlate linearly with clinical severity. An additional important point concerning the enzymatic findings in our case is that the initial lysosomal screening at 11 months performed on a dried blood spot was reported as normal except for elevated hexosylsphingosine. The subsequent detection of β-galactosidase deficiency likely reflects the use of a different, more sensitive assay method in leukocytes and/or the progressive accumulation of substrate over time, which may unmask a borderline enzyme deficiency that was not apparent earlier.

Collectively, these findings provide further support for the role of codon 270 as a potential critical functional site within *GLB1*, where even subtle amino acid substitutions can result in severe enzymatic dysfunction and early-onset neurodegeneration.

## 5. Conclusions

GM1 gangliosidosis is a severe and progressive lysosomal disorder driven by β-galactosidase deficiency, ultimately leading to profound neurodegeneration. The p.Tyr270Asp (c.808T>G) variant is consistently associated with the infantile form of the disease and with marked impairment of enzymatic function, reflecting the critical role of the tyrosine residue at position 270 in maintaining catalytic activity.

Evidence from published clinical cases indicates that this variant is linked to a severe neurodegenerative phenotype characterized by early developmental delay, progressive regression of motor skills, and multisystem involvement, including hepatosplenomegaly, cardiomyopathy, skeletal abnormalities, facial dysmorphism, and the retinal “cherry-red spot”.

The present case further supports these genotype–phenotype correlations while expanding the mutational spectrum of *GLB1*, describing for the first time a compound heterozygous state involving two variants affecting the same codon (c.808T>G/c.808T>C).

However, it is important to note that codon 270 is not exclusively associated with GM1 gangliosidosis; rather, it lies within the broader spectrum of *GLB1*-related disorders, which also includes mucopolysaccharidosis IVB (Morquio B disease). Collectively, these findings provide support for the theory of codon 270 as a potential critical functional site within *GLB1*, where even distinct amino acid substitutions can result in severe enzymatic dysfunction and an early-onset neurodegenerative course. Further reports and functional studies are needed to fully define the genotype–phenotype correlation at this site.

## Figures and Tables

**Figure 1:**
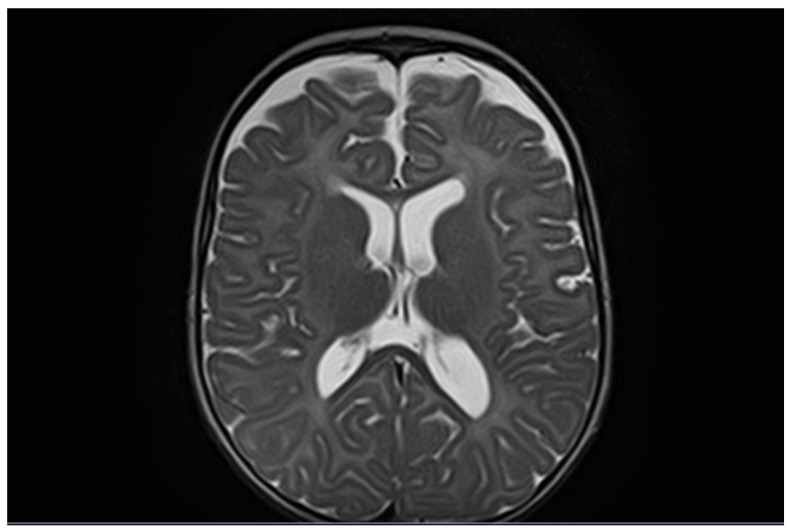
Patient K. Brain MRI at 11 months (1.5 Tesla, AX T2).

**Figure 2:**
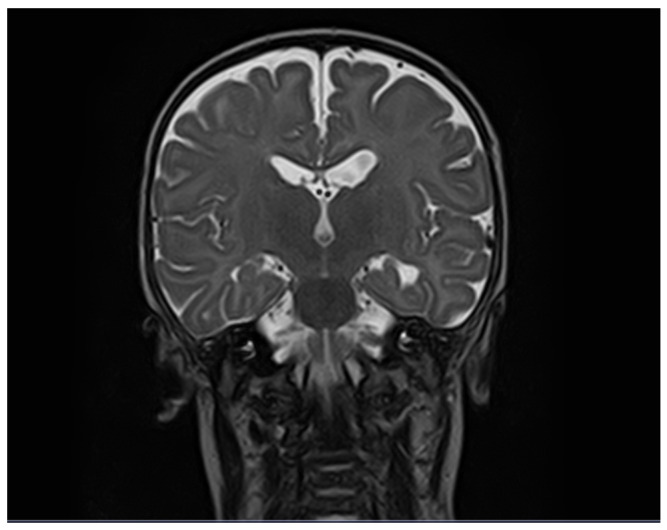
Patient K. Brain MRI at 11 months (1.5 Tesla, CO T2). MRI—signs of delayed myelination processes, mild periventricular leukomalacia, likely secondary to hypoxic–ischemic injury, and mild hypoplasia of the cerebellum and corpus callosum.

**Figure 3:**
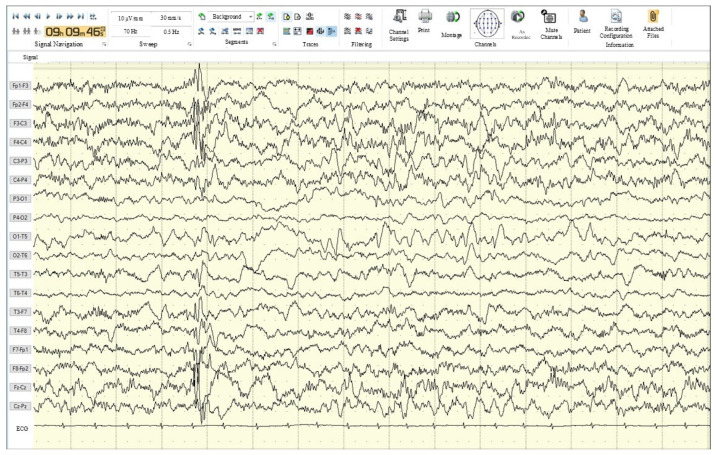
Patient K. EEG at 2 years. Sleep. Note: 100. μV), interictal regional epileptiform discharges polyspike-slow wave predominantly in the frontal-central areas, alternating lateralization, mostly from the right.

**Figure 4:**
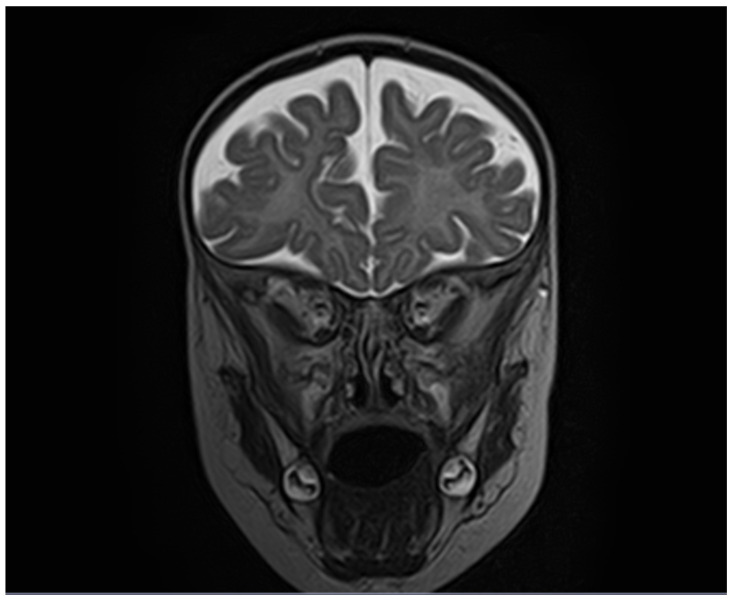
Patient K. Brain MRI at 2 years (1.5 Tesla, CO T2): MRI—signs of progressive atrophy of the cerebral hemispheres.

**Table 1:** Comparative analysis of clinical and genetic characteristics associated with variant NM_000404.4(*GLB1*):c.808T>G (p.Tyr270Asp) according to scientific literature data.

№	Link to the Source	Number of Reported Cases	Number of Patients with Nucleotide Variant c.808T>G in Genotype	Patients’ Genotype	Note
1.	Paschke E et al., 2001 [34]	17	1	p.Thr82Met/p.Tyr270Asp	c.808T>G (p.Y270D) was first described as a novel mutation in one patient in a compound heterozygous state
2.	Hofer D et al., 2009 [35]	20	5	p.Tyr270Asp/p.Tyr270Asp; p.Tyr270Asp/p.Tyr270Asp; p.Tyr270Asp/c.1601_1602insGCCA; p.Tyr270Asp/p.Lys346Asn; p.Tyr270Asp/p.Thr82Met.	c.808T>G was detected in 7 of 50 alleles (14%) and was identified by the authors as a frequent mutation in the *GLB1* gene in patients with GM1-gangliosidosis
3.	Higaki K et al., 2011 [36]	26	1	p.Arg201Cys/p.Tyr270Asp	
4.	Rozenval’d, I.E et al., 2021 [37]	1	1	p.Tyr270Asp/p.Lys346Asn	
5.	Bychkov I et al., 2022 [38]	1	1	p.Tyr270Asp/PP NPM1 in the intron 5 of *GLB1*	A rare variant in the compound heterozygous state is a combination of a missense mutation and the insertion of a processed pseudogene from the retrotransposon group into the *GLB1* gene.
6.	Description of the authors’ clinical case of patient D., 3 years old	1	1	p.Tyr270 Asp/p.Tyr270 His	

**Table 2:** Comparative characteristics of patients with GM1-gangliosidosis and mucopolysaccharidosis IVB with nucleotide variant c.808T>G (p.Tyr270Asp) in *GLB1*.

№	Genotype	Clinical Phenotype	Age of Manifestation	Age of Diagnosis	NS	CRS	CI	HSM	SA	Beta-Galactosidase *	Ethnic Background	Reference
1	p.Thr82Met/p.Tyr270Asp	Morquio disease type B	2 years	4 years	+	n. r.	n. r.	n. r.	+	↓/0.23 nmol/mg min	German	[34]
2	p.Tyr270Asp/p.Tyr270Asp	GM1-gangliosidosis (type 1)	6 months	1 year	n. r.	n. r.	n. r.	+	+	↓/2%	Bosnian	[35]
3	p.Tyr270Asp/p.Tyr270Asp	GM1-gangliosidosis (type 1)	2 months	11 months	+	+	−	−	+	↓/2.5%	Czech	
4	p.Tyr270Asp/c.1601_1602insGCCA	GM1-gangliosidosis (type 1)	2 months	1 year 6 months	+	n. r.	n. r.	+	+	n. r.	Hungarian	
5	p.Tyr270Asp/p.Lys346Asn	GM1-gangliosidosis (type 1)	4 months	7 months	+	+	−	+	+	↓/3.4%	Czech	
6	p.Tyr270Asp/p.Thr82Met	GM1-gangliosidosis (type 3)	2 years	4 years	+	−	−	−	+	↓/3.4%	German	
7	p.Arg201Cys/p.Tyr270Asp	GM1-gangliosidosis (type 1)	n. r.	n. r.	n. r.	n. r.	n. r.	n. r.	n. r.	n. r.	Estonian	[36]
8	p.Tyr270Asp/p.Lys346Asn	GM1-gangliosidosis (type 1)	6 months	1 year 6 months	+	−	n. r.	+	+	↓/2.5 nM/mL/h	n. r.	[37]
9	p.Tyr270Asp/PP NPM1 in the intron 5 of *GLB1*	Morquio B disease	6 years 10 months	9 years	n. r.	n. r.	n. r.	n. r.	+	↓/(0.72 0.4 nM/mL/h	n. r.	[38]
10	p.Tyr270 Asp/p.Tyr270 His	GM1-gangliosidosis (type 1)	6 months	1 year 6 months	+	+	+	−	+	↓/27.73 µM/L/h	Russian	Current paper

* The β-galactosidase activity values are reported in the units as originally provided by the authors of the cited studies. The downward arrow (↓) indicates a decrease in β-galactosidase activity compared with the respective reference range for the specimen type and laboratory. Note. +, clinical feature present; −, clinical feature absent.

## Data Availability

The original contributions presented in this study are included in the article. Further inquiries can be directed to the corresponding author.

## Abbreviations

The following abbreviations are used in this manuscript:

NS	neurological symptoms
CRS	cherry-red spot
CI	cardiac involvement
HSM	hepatosplenomegaly
SA	skeletal abnormalities
n. r.	not reported
